# Ordered fragmentation of oxide thin films at submicron scale

**DOI:** 10.1038/ncomms13148

**Published:** 2016-10-17

**Authors:** L. Guo, Y. Ren, L. Y. Kong, W. K. Chim, S. Y. Chiam

**Affiliations:** 1 Department of Electrical and Computer Engineering, National University of Singapore, 4 Engineering Drive 3, Singapore 117583, Singapore; 2 Institute of Materials Research and Engineering, A*STAR (Agency for Science, Technology and Research), 2 Fusionopolis Way, Innovis 138634, Singapore; 3 NUS Graduate School for Integrative Sciences and Engineering, National University of Singapore, 28 Medical Drive, Singapore 117456, Singapore

## Abstract

Crack formation is typically undesirable as it represents mechanical failure that compromises strength and integrity. Recently, there have also been numerous attempts to control crack formation in materials with the aim to prevent or isolate crack propagation. In this work, we utilize fragmentation, at submicron and nanometre scales, to create ordered metal oxide film coatings. We introduce a simple method to create modified films using electroplating on a prepatterned substrate. The modified films undergo preferential fragmentation at locations defined by the initial structures on the substrate, yielding ordered structures. In thicker films, some randomness in the characteristic sizes of the fragments is introduced due to competition between crack propagation and crack creation. The method presented allows patterning of metal oxide films over relatively large areas by controlling the fragmentation process. We demonstrate use of the method to fabricate high-performance electrochromic structures, yielding good coloration contrast and high coloration efficiency.

## Abstract (web summary)

Fracture and related processes are typically considered detrimental, but have also attracted interest in more constructive roles. Here authors demonstrate ordered fragmentation at submicron scales of a metal oxide/hydroxide thin film by introducing preferential sites for fracture on the underlying substrate.

## Graphical abstract

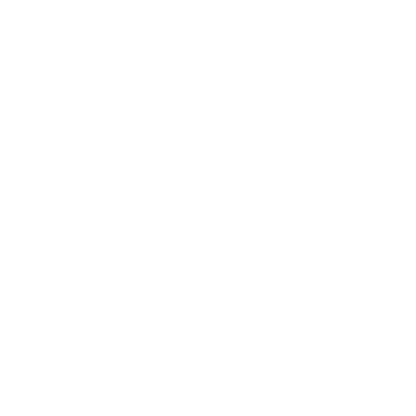

Nanostructuring of materials is a promising approach to enhance performances of any surface driven phenomenon such as electrochemical, catalytic, sensing or energy related technologies^1^,^2^,^3^,^4^,^5^,^6^,^7^. There have been various approaches towards structuring the materials but many of these can be largely classified as template assisted phenomena. These include template assisted nano-Kirkendall effect^8^, template assisted phase separation^9^, template assisted dewetting^10^,^11^,^12^ and template assisted evaporative assembly^13^. In this work, we introduce a new approach towards nanostructuring by utilizing template assisted fragmentation in thin films. Fragmentation is typically viewed as an undesirable failure mechanism. However, this has also been utilized to create semi-regular patterns in crackle for celadon ceramics^14^,^15^,^16^. The ability to control such fragmentation process is difficult and previous attempts have investigated the use of magnetic fields^17^, electric fields^18^, directional drying^19^ and more commonly, creation of micro-notches^20^,^21^,^22^. While the use of micro-notches was highly successful in controlling the cracks, there are some limitations. It can be cumbersome to create regular patterns with the micro-notches since these are typically created individually. Another major drawback is the limitation in dimensions. While a small crack width of ∼500 nm is possible^22^, the distance between crack lines are restricted by the size of the micro-notches and this ranges from 5 to 50 μm (ref. 21). The ability to induce ordered fragmentation at the submicron scale is difficult. Here, we introduce a method that allows for such fragmentation control. The approach targets ordering of the starting material, such that fragmentations are preferred in certain regions of the film. The resultant nanostructured film coatings are unique, and not easily obtained otherwise. In this work, we choose to examine a technologically relevant material, nickel oxide/nickel hydroxide or NiO/Ni(OH)_2_, which is popular in electrochemical devices due to the low cost, superior specific capacity and excellent anodic coloration efficiency^8^,^23^,^24^. Nanostructuring of NiO/Ni(OH)_2_ is important as this brings about substantial enhancement to the performance, which have been demonstrated for a variety of structures, including porous thin films^25^, wires^26^, tubes^27^, core/shell^28^ or 3D hierarchical gyroids^7^.

The fragmentation phenomenon, in general, has attracted many experimental and theoretical studies that investigated the complex geometry, sizes and distribution of the fragmented patterns^29^,^30^,^31^,^32^. Such investigations are relevant in understanding many natural occurring fragments such as skin, fruits, wood, paint and ceramics. More importantly, these models provided important predictive capabilities in examining and limiting the failure of materials^33^,^34^,^35^,^36^. One such fragmentation process controls the applied stresses from a change in the volume before and after a drying process^37^,^38^. A typical example from nature is the mud-cracking phenomenon, whereby fragments of irregular shapes and sizes are formed on the surfaces after the top layer shrinkage^39^,^40^. This behaves similarly for the drying of thin film coatings, whereby the rigid substrate acts as a constraining bottom layer. During desiccation, under the compressive strain induced by the volume change of the thin film, bi-axial tensile stresses are applied to weak spots in the film. Cracks will form when the resultant strain builds up beyond a critical value.

In this work, we utilize this mud-cracking concept to produce ordered fragments at the submicron scale using an artificially modified thin film. Distinct weak spots that have a propensity for forming cracks are created. The obtained structures, after the drying process, are otherwise not possible in any natural occurring fragmentation. We have also tested the electrochromic performance of such nanostructured nickel oxide coatings where some compelling results are demonstrated. The specific implementation discussed here combines a lithographic method to generate weak spots, electrodeposition to introduce the initial oxide film and water loss on drying to drive fragmentation, but it may be possible to generalize the overall approach to other materials and processes, while preserving the ability to pattern arrays of submicron features over large areas.

## Results

### Creating a modified film

Fragmentation is a strain relief process that lowers the free energy of the material^41^. Statistical models are often used because of the stochastic nature of fragmentation processes. However, we will show that fragmentation can be controlled to some extent if regions of lower mechanical strength can be introduced. Here, we propose and show that this can be achieved by fabricating a thin film that is attached to a rigid substrate through a regular array of pillars. While this sounds like an unconventional and complicated structure, it is actually quite natural when a template assisted electroplating approach is used. The choice of the templates and the control of the electroplating will be the critical governing parameters in creating different structures.

**Figure 1:**
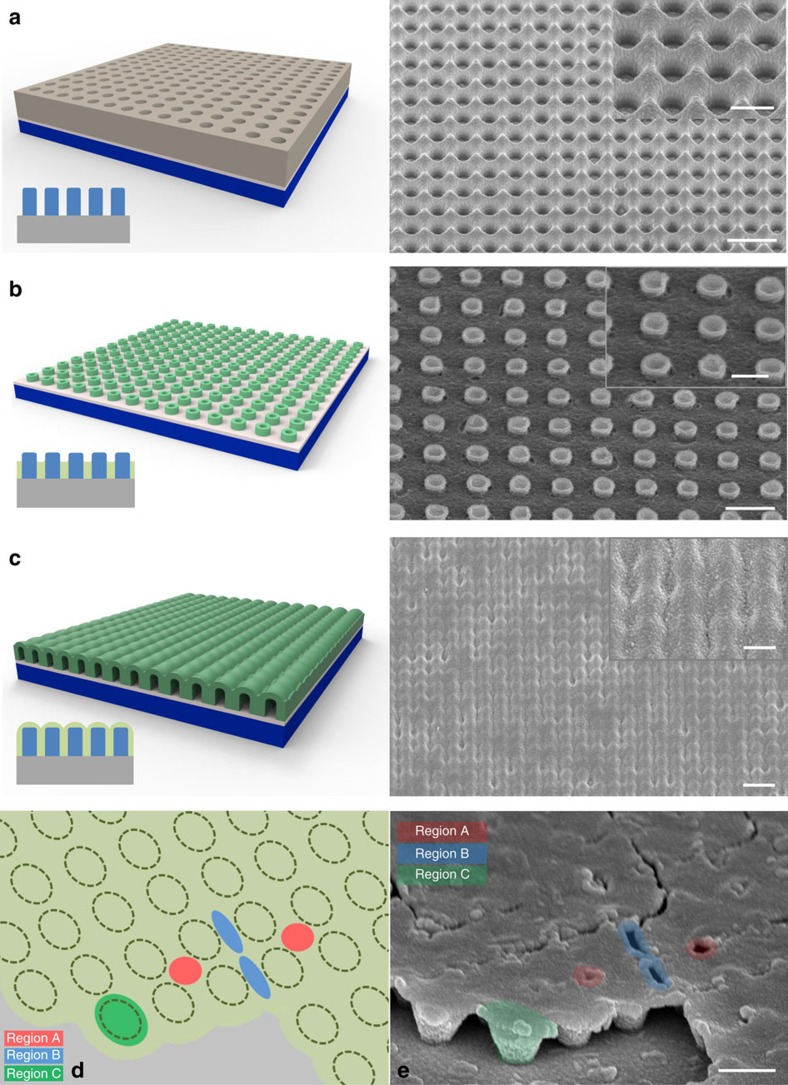
Fabrication of modified film. Schematic and SEM images for (**a**) laser interference lithography fabricated templates. (**b**) Formation of nanopillars after a short plating duration. (**c**) Formation of continuous overgrowth layer with long plating duration. (**d**) Schematic diagram and (**e**) SEM image of air-dried films showing representative regions of A, B and C of a modified film. These highlighted regions represent locations with different propensity towards crack formation, with Region A being the weakest, followed by Region B, and then Region C. All scale bars are 1 μm in the main images (**a**–**c**), and 500 nm in the inset images and in **e**.

The templates we utilize are created from laser interference lithography (LIL). The schematics of such templates are shown in Fig. 1a, with corresponding scanning electron microscopy (SEM) images. During the plating process, growth proceeds from the substrate, whereby pillars are first formed, as shown in Fig. 1b. Further increment of the plating time can eventually yield a continuous (though not necessarily flat) film, as shown in Fig. 1c. The thin film overgrowth is uniquely different from typical film coatings. After the removal of the photoresist, the structure can be viewed as a suspended thin film supported by a regular array of pillars. Figure 1d shows a schematic of such a modified thin film with representative regions indicated as A, B and C. These highlighted regions represent areas with different propensity towards crack formation. Region A has the least amount of overgrowth since it is farthest away from the plating holes. Being farther away from the holes also necessarily yields a higher residual photoresist, a natural consequence of the LIL process (Fig. 1a). Region A is also an area whereby the film has absolutely no support from the pillars. A thin overgrowth layer, without any anchoring to the rigid substrate, makes it mechanically weak. All these factors make Region A the weakest spot that is prone to crack formation. Comparatively, the suspended Region B will have a stronger resistance to cracking, since it is nearer to the plating holes with a thicker amount of overgrowth. Finally, Region C is the strongest spot as there are underlying pillars that are anchored to the substrate.

The accompanying SEM micrograph in Fig. 1e shows an example of the described hierarchy between the three regions. The micrograph shows a thin film overgrowth of 115 nm, after a simple air drying process that can represent the onset of crack formation. This is because such air drying does not induce the loss of structural water or any phase change and thus, the volume change and stress build-up are significantly smaller^42^,^43^. We also note that our drying process also does not induce significant capillary forces since drying of the thinner overgrowth does not induce crack formation^44^. The SEM image in Fig. 1e shows clearly the crack formation, or the onset of crack formation, in most of the Region A. Some crack formation and propagation can also be observed along Region B, while none are detected in Region C. This clearly demonstrates the success of creating a modified thin film with ordered regions of different propensity for crack formation.

### Fragmentation of modified films

**Figure 2:**
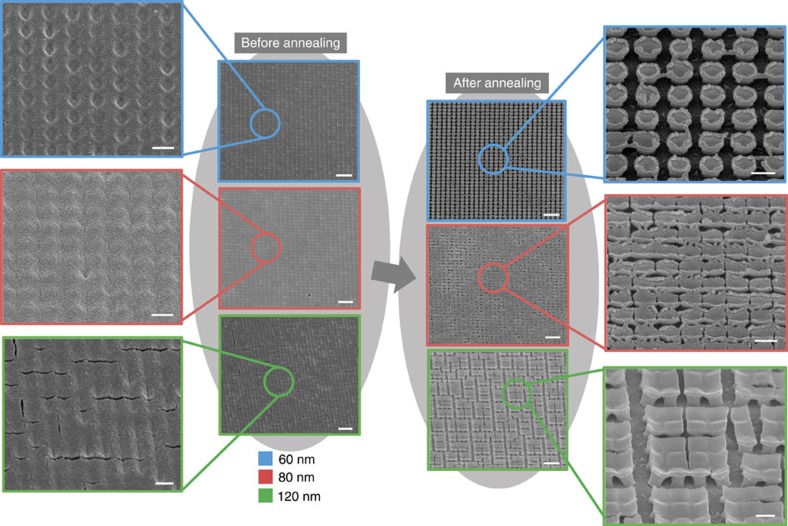
Fragmentation of modified film. SEM images of of Ni(OH)_2_ thin film overgrowths for indicated thicknesses of 60, 80 and 120 nm, before and after thermal annealing. A magnified SEM image for the structures after annealing is also shown for each thickness. All scale bars are 2 μm in the main images and 500 nm in the magnified images.

Having created the weak spots in the modified thin film, we can test the fragmentation process at different overgrowth thicknesses after a furnace anneal at 300 °C for 90 min. This processing condition is expected to introduce significant stresses to the films as discussed in the Methods section. SEM images of thin film overgrowth, before and after annealing, for the indicated thicknesses (plating duration) of 60±10 nm (20 s), 80±15 nm (60 s) and 120±20 nm (100 s), are shown in Fig. 2. Magnified images of each film before and after annealing are also shown. We note here that the chemical compositions of the created structures are essentially similar for the different thicknesses as determined by X-ray photoelectron spectroscopy (Supplementary Fig. 1, Supplementary Table 1 and Supplementary Note 1). The coated films also remain amorphous after the heat treatment as determined by X-ray diffraction (Supplementary Fig. 2 and Supplementary Note 2). For an overgrowth of 60 nm, a continuous coverage is observed before the annealing. After the thermal treatment, only the anchoring pillars remain. A more detailed observation of the pillars actually shows some crinkle formation and this can be explained by the rate of water loss from the surface, or a hindrance effect (Supplementary Fig. 3a,b and Supplementary Note 3). It is useful to understand that the resultant structures actually demonstrated the hierarchy in crack formation we previously described. Apart from the obvious observation that Region C shows the highest stability, the order of stability for Regions A and B can be derived from the observation of remaining bridging materials only for Region B. When a thicker overgrowth of 80 nm is annealed, the film shows a different transformation. It can be seen that after annealing, the structured film yielded well-ordered cracks, without any clear crack propagation. These generated cracks without any crack propagations are in the nanoscale regime, similar to those formed from initial air-drying. The individual pillars can still be discerned even though they no longer stand out clearly. The edge crinkle effect is also less obvious and this gives such films good coverage, whereby the underlying substrate is not greatly exposed. We will show subsequently that such a structure actually yields the best electrochromic performance. Finally, for the thickest overgrowth of ∼120 nm, some crack formations are present even before the furnace drying process. This is similar to the air-dried film, as shown in Fig. 1e. At such thickness, the film is susceptible to crack formation even with air drying due to the larger stress built up.

After the furnace anneal, the SEM image for the 120 nm overgrowth thickness sample shows a lower number of crack formations with clear signs of crack propagation. Propagation of cracks now appears to be a competitive way to relieve the strain as opposed to creating new cracks for such thick films. This agrees with theoretical and experimental findings of larger crack spacing and fragment size that are previously reported as thickness of the film increases^40^,^45^,^46^,^47^. As a result, larger repeated structures are formed instead. These repeated structures for the 120 nm overgrowth thickness sample shows typical sizes spanning across two pillars in orthogonal directions; we hereby refer to such units as 2 × 2 structures for easy reference. These structures are highly characteristic and a low magnification image (Supplementary Fig. 4) shows the interesting patterns derived over a large area (Supplementary Note 4). A high magnification image also reveals the presence of the crinkle effect for these large units via a similar formation process (Supplementary Fig. 5a). The crinkle effect of the larger structures shows possible concurrent film cracking (Supplementary Fig. 5b) and the delamination of the pillars (Supplementary Note 5). This can represent comparable strain relief energies between the adhesion at the hetero-interface and the creation of a crack in a homogenous material. Such studies might be a viable approach to study the adhesion of nanostructures if the mechanical properties of nanomaterials are known^48^.

**Figure 3:**
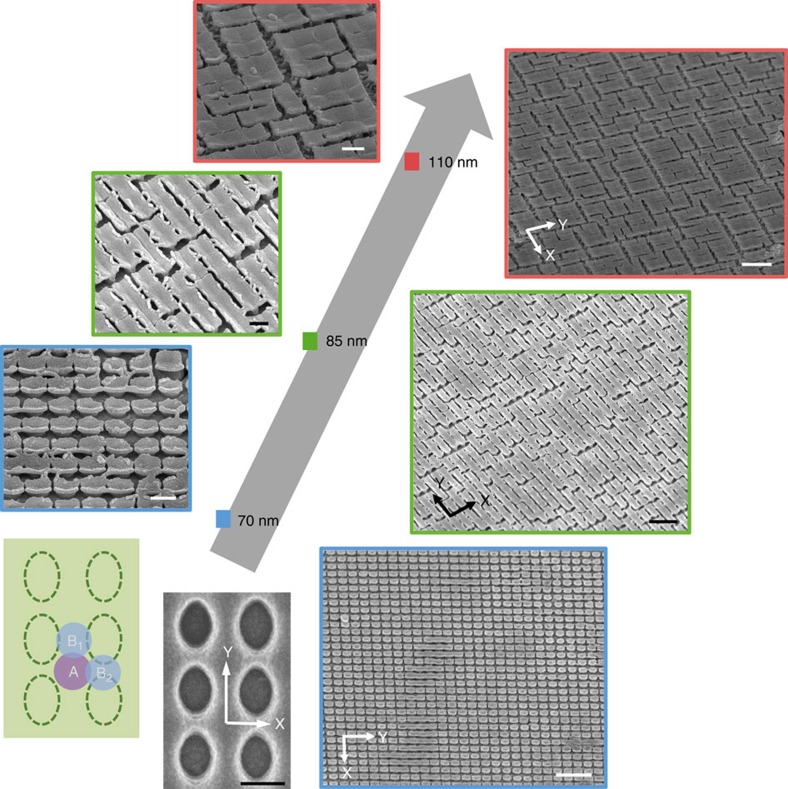
Fragmentation of anisotropic patterns. SEM images for different thickness of overgrowth (70, 85 and 110 nm) plated using an oval shaped template. A schematic of the oval shaped template showing the representative area (A, B_1_ and B_2_) is also shown together with the SEM micrograph depicting the anisotropic direction *Y* and *X*. The corresponding direction *Y* and *X* are also shown in the SEM images at the bottom. All scale bars are 2 μm in the images on the right and 500 nm in the magnified images on the left.

The reported structures achieved via the templated plating and fragmentation are unique and not easily achievable otherwise. The resultant structure of a suspended film structure with regularly populated cracks fabricated using our proposed method cannot be easily achieved via direct lithography. In addition, different overgrowth thickness and the eventual structures obtained demonstrated the control we have over the fragmentation process, and such an approach of fabrication has some advantages. The structures are essentially shaped by the templates and with advanced lithography techniques, the method will allow for the formation of fragments at the nanoscale dimension. It is also quite simple to utilize our understanding in the creation of such weak spots to synthesize other characteristic structures. In Fig. 3, we show the fabrication of anisotropic patterns using oval shaped hole templates and the subsequent SEM images obtained for different thickness of overgrowth. It is important to realize that for such anisotropic patterns, Region A remains the weakest spot. However, due to the anisotropy in the distance from the plating holes, slight differences exist between Regions B1 and B2. The reduced spacing between neighbouring holes and the lower photoresist height, will lead to an increase in overgrowth thickness in Region B2 as compared with Region B1. Due to this anisotropy, we expect cracks to form preferentially in Region B1, and along the *Y*-direction as indicated in the accompanying SEM image. Having accumulated more cracks along the *Y*-direction, we also expect a greater probability in crack propagation and fragmentation lines. The resultant effect of such fragmentation preference is to form more rectangular like structures, as opposed to the more square shaped isotropic structures when round hole pattern templates are used. Therefore, Fig. 3 shows the formation of oval shaped structures for the 70±10 nm overgrowth thickness sample. Such oval shaped pillars remain connected in Region B2 as shown in the accompanying SEM image at the top. When the overgrowth thickness is increased to 85±15 nm, there is a tendency in forming rectangular-like structures after the drying process. The longer side of the rectangle, as defined by the template, is in the *Y*-direction as indicated in the SEM image. Finally, for an overgrowth thickness of 110±20 nm, larger rectangular shaped structures are observed. This is similar in nature to the isotropic structures in having less crack creation and more crack propagation. Judging from the resultant structures created for all the overgrowth thicknesses, the anisotropic patterns have successfully altered preferential crack formation and propagation, whereby characteristic rectangular-like nanostructures are obtained.

### Fragmentation process of modified films

**Figure 4:**
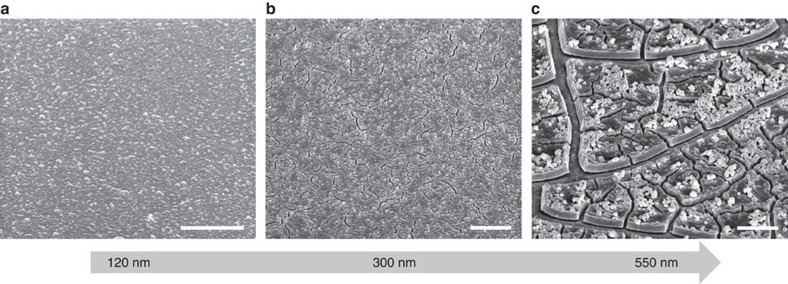
Fragmentation of non-patterned thin films. SEM images of non-templated electroplated Ni(OH)_2_ thin films for indicated thickness (**a**) 120 nm, (**b**) 300 nm and (**c**) 550 nm after thermal annealing treatment. All scale bars are 2 μm in the images.

It is important to further understand the formation process for such modified (templated) thin films. The modified films are anchored to a rigid substrate through the base of the pillar. Such a configuration is necessary for early and controlled fragmentation. We emphasize that the obtained structures demonstrated are otherwise not possible for a coated thin film. Firstly, the critical thickness for crack formation of a coated thin film is larger. Figure 4 shows the SEM images of increasing thickness of a non-templated plated thin film, using the same electroplating method, after the furnace drying process. At 120 nm, there is no observable crack formation, which is in contrast to the routine fragmentation of modified films (∼60–120 nm thickness), as shown in Figs 2 and 3. Crack formation is only observed for the non-templated film when the thickness is increased to ∼300 nm. This is a consequence of the higher built-up strain energy for the thicker film. Finally, when the thickness is increased to ∼550 nm, crack propagation similarly becomes an important strain relief mechanism with less cracks being created. However, unlike the modified thin film, the fragments formed are much more random in nature and fragments of varying sizes and shapes are obtained. If we estimate the critical thickness for the thin film to be ∼200 nm, we can comment on the fracture toughness (*K*_IC_) of the coated film. Simplifying the equation for critical thickness (*h*_c_) to: *h*_c_=2.49*K*^2^_IC_*E*^−2^*ɛ*^−2^, where we have taken the Poisson's ratio to be 0.3 and a dimensionless parameter of ∼1.15 for a crack geometry of 1:3, *K*_IC_ can be calculated with known values of Young modulus (*E*) (refs 37, 49). Reported Young modulus values of NiO are around 5.2 × 10^10^ to 10^11^ Nm^−2^ (refs 50, 51). Using these values, we calculate the fracture toughness to be 2.2–4.3 M Nm^−2^. This is within reasonable expectations for ceramics^52^, but higher than the reported values for NiO obtained using modelling estimates^53^. We note that the modelling estimates appears to underestimate fracture toughness of materials such as Al_2_O_3_ and TiO_2_^54^,^55^. Using the same calculation, the modified thin film reduced this fracture toughness to <1.2–2.3 M Nm^−2^, thereby effectively lowering the critical thickness. We attribute this difference to primarily the adhesion of a thin film on a rigid substrate. In a typical thin film coating, when tensile stress is exerted on the film, compressive forces within the substrate counter this force and this is applied to the film through its adhesion to the rigid substrate^47^,^56^. The effective fracture toughness of the suspended film can thus be lowered as this additional resistance from the substrate is absent, causing early fragmentation of the mechanically weak spots as shown.

### Statistical analysis of fragments

**Figure 5:**
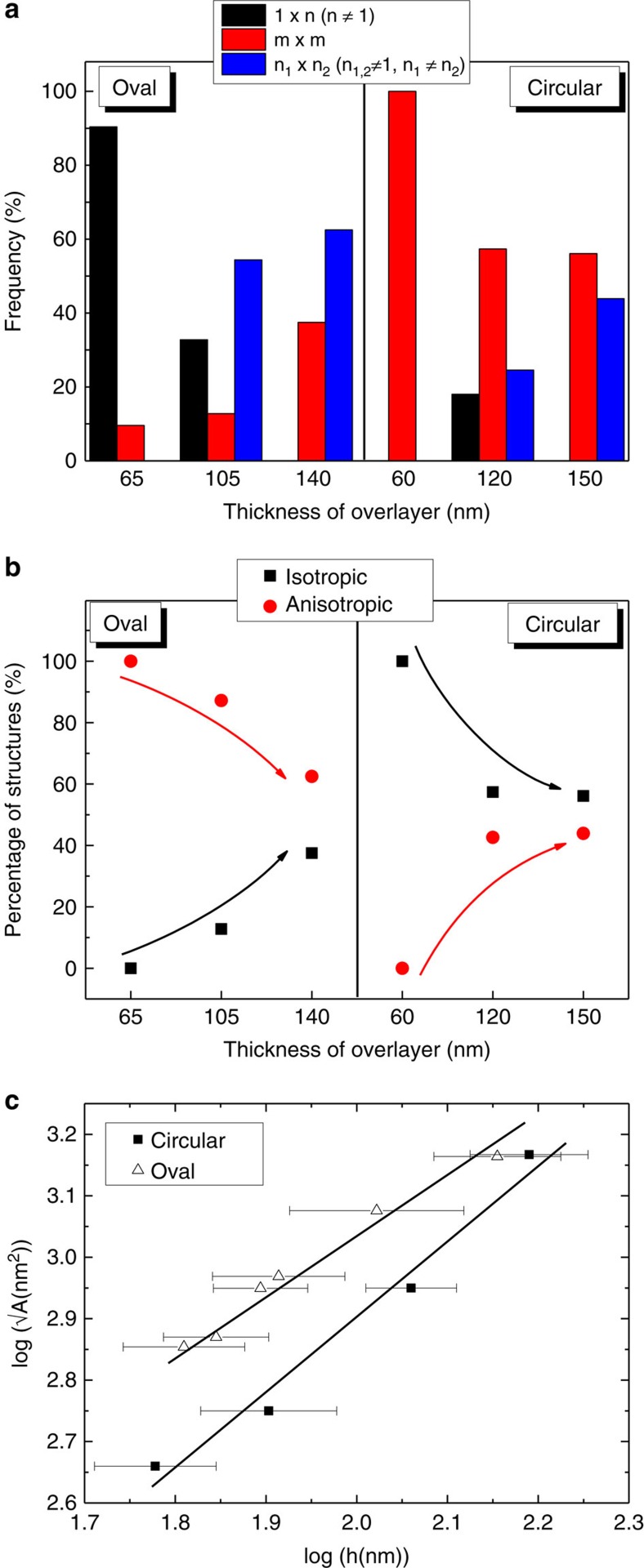
Statistical analysis of fragments. (**a**) Summarized statistical results of fragment size distribution for circular and oval patterns for different overlayer thicknesses. The fragment size were classified into 1 × n, m × m, n_1_ × n_2_, representing single rectangular fragments (1 × 2, 1 × 3, 1 × 4 and so on), squares (1 × 1, 2 × 2, 3 × 3 and so on) and larger rectangular fragments (2 × 3, 3 × 4, 2 × 4 and so on). (**b**) Summarized plots of isotropic versus anisotropic fragments for both circular and oval patterns for different overlayer thicknesses. (**c**) Dependence of the average square root area of fragments (√A(nm^2^)) on the thickness (h(nm)) of the modified film. Triangles and squares correspond to samples fabricated using templates of oval and circular patterns, respectively. Error bars for deviation of thickness shown are s.d.s obtained from the measured thicknesses. The error bar for the fragment sizes is not shown as these are relatively small.

Despite using a modified thin film for ordered fragmentation, a more detailed statistical analysis shows some similarity in trends, perhaps suggesting the same governing physics in such processes. The fabricated structures are distinctively characteristic, but they are not perfectly uniform units, especially for thicker overgrowths. For the thinner overgrowths as seen for both the isotropic and anisotropic patterns, regular building block sizes, as dictated by the pillars, can be obtained (1 × 1 structure). This is because the fragmentation process here is confined mainly to crack initiation and the modified thin film gives excellent uniformity. For thicker modified films, crack propagation comes into play. When this happens, there will be some randomness to the formation of the fragments. A summarized distribution of fragments for the different overlayer thicknesses is shown as histogram plots in Fig. 5a. The distribution for the optimized thicknesses are not shown, because it is not straight forward to define fragment sizes for films that are only populated with cracks. Instead, we included distribution for even thicker overlayers where the crack propagation dominates. The distribution is divided into structure sizes of 1 × *n*, *m* × *m* and *n*_*1*_ × *n*_*2*_, representing fragment sizes of single unit rectangles, squares and larger rectangles, respectively. Figure 5a shows clear trends in the fragment size distribution for both the oval and circular patterns as the overlayer thickness is increased. For the oval patterns, the intended rectangular structures clearly dominate at lower overlayer thicknesses. When the randomness of the crack propagation sets in, there is a shift towards formation of larger rectangular or even square structures. Similarly for the circular patterns, the intended square fragments dominate before seeing the increased contribution from rectangular structures for thicker overlayers.

A summary of the relative make up of isotropic and anisotropic fragments is shown in Fig. 5b. It is immediately clear that the oval and circular patterns show distinctive ability to create the intended isotropicity for the fragments, even when crack propagation sets in for the thicker overlayers. It is also clear that as the overlayer thickness increases, the isotropic and anisotropic fragment distributions tend towards a more random and hence equal distribution. Nonetheless, the respective patterns still kept an asymmetric distribution (∼60:40) even for the thickest overlayer investigated in this work. This indicates the success of using the respective structures in guiding the isotropicity nature of the crack formation. A summary statistics for the variation of the fragment sizes with all the range of film thicknesses, is shown in Fig. 5c for both the circular and oval shaped pattern films. It can be seen that the log–log plot of the square root of average fragment size (*A*) versus thin film thickness (*h*) yields a linear relationship. Scaling sizes of fragments is shown to be quite universally dependent on substrate properties, such as the number of bonds per block unit^46^. This was also demonstrated experimentally, where the linear scaling law of √*A*=*Kh*^*n*^ (for *n*=1), with dimensionless ratio *K*, was found for isotropic and directional crack patterns for variation of thickness in coated Fe_2_O_3_ nanocrystals^45^. Therefore, it may be intuitive that even for the modified thin films, similar scaling law governs the mechanics of crack formation. The log–log plots for the oval and circular patterns can both be fitted with linear profiles. For the oval patterns, the linear scaling law appears to still apply with a fitted *n* value of ∼0.93. The proportionality constant (*K* value) is larger than that reported for the coated Fe_2_O_3_ at ∼15.1 and this can be explained by the lowering of fracture toughness of the modified films. Interestingly, for the circular patterns, the scaling law deviates from the linear dependence with a larger *n* value of ∼1.24. The corresponding *K* value for the circular pattern is lower at 2.68. On the basis of the equation, the *K* value reflects the size of the fragments at lower thicknesses, while the *n* value characterizes the scaling. Thus at lower thicknesses, we should expect circular patterns to yield smaller fragments. This can be directly attributed to a greater tendency for crack propagation for the oval structures, while crack creation is the dominant mechanism for the circular structures. As for the scaling capacity for higher thickness where crack propagation is rampant, we propose that the meeting of propagating cracks contributes an additional factor. This happens more rarely when the cracks propagate preferably in parallel directions, as was the case for the oval shaped patterns. For the circular patterns, isolation of some fragments occurs when propagating cracks meet, giving an additional mechanism in creating larger structures. This explains the higher scaling relationship observed. Such understanding or statistics presents a facile approach for creating semi-regular nanostructures through fragmentation in a modified thin film. Characteristic sizes can therefore be achieved just by selection of templates with the required pattern and the control of overgrowth thicknesses. For example, a typical fragment size of ∼0.3 μm^2^ can be obtained using a circular pattern template with an 80 nm film overgrowth. A switch to the oval pattern template will yield a larger fragment size of ∼0.8 μm^2^. We believe that larger or smaller sizes extrapolated from the plot can also be achieved and the possibility is limited by the practicability of fabricating the respective templates.

### Electrochromic performance of structured films

**Figure 6:**
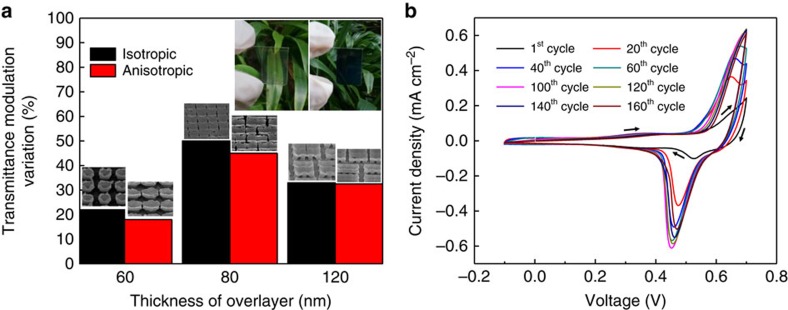
Electrochromic performance of structures. (**a**) Plot of transmittance modulation variation versus overlayer thickness for Ni(OH)_2_ deposited in photoresist templates with circular and oval patterns. The magnified SEM image above each column shows the surface morphology of each structure. Inset shows an example of the obtained bleached and colour state for the structured film. (**b**) Cyclic voltammetry characteristics of Ni(OH)_2_ deposited in photoresist template with oval shaped hole pattern with a 80 nm overlayer thickness, recorded at a scan rate of 10 mV s^−1^ up to 160 cycles. The arrows indicate the direction of the sweep during the cyclic voltammetry measurements.

Electrochromic (EC) performance of a material, like all electrochemical devices, is strongly affected by its surface area and accessibility to the electrolyte. In addition, EC devices have the added constraint of having to provide sufficient optical contrast. The latter point requires suitable morphology and coverage of a coating. Such a requirement makes our structure especially relevant in the study of EC materials. *In-situ* transmittance modulations are performed with cyclic voltammetry (CV) measurements to investigate the switching and optical properties of the obtained NiO structures. There are no significant differences to the shape or peak potential of the CV curves obtained between the structured film of different overlayer thicknesses (Supplementary Fig. 6 and Supplementary Note 6). The current density, however, varies as this is related to the reactivity and the optical performance. The summarized transmittance modulation for the different representative structures, are shown in Fig. 6a, where a picture showing an example of the bleached and coloured EC state is included in the inset. The lowest transmittance modulation is observed for the regular pillars array structure. This can be attributed to the lack of coverage, thereby yielding a low optical contrast. For the structures with sufficient coverage, a greater number of cracks will expectedly be preferred since this provides a better accessibility of the electrolyte for the material. Therefore, structures with a high number of well distributed cracks, for example that of the 80 nm overlayer thickness plated film gives a higher contrast when compared with thicker samples with 120 nm overlayer thickness. The thicker films with a smaller number of cracks and larger fragment sizes experience a slight drop in the optical contrast. It is important to note that without structuring, thin films of comparable amount of materials used yield a transmittance modulation of only ∼18.5%, while the nanostructured film can easily achieve values of 40% or more. This demonstrates that high-performance EC devices can possibly be achieved using a structuring approach of coated films. The coloration efficiency (CE) for the structured films can also be compared. The CE is a figure-of-merit to judge the efficiency of the EC material by examining the optical contrast per unit charge. The CE is defined as Δ*OD*/*Q*, where the optical density modulation (Δ*OD*) describes the optical contrast (that is, ln(*T*_bleached_/*T*_colored_)) and *Q* is the charge density^57^. The CE values of different structures are calculated and listed in Table 1, together with CE values from other reports^8^,^26^,^58^. Table 1 shows that our structured NiO/Ni(OH)_2_ demonstrated one of the highest reported CE values as compared with reported nanostructured materials with a value of greater than 70 C^−1^ cm^2^. The high CE value is an interesting finding. Even though the chemical composition and microstructures can affect the performance of an electrochromic material^59^, we have shown in earlier discussions that this does not vary greatly across the different created structures and hence the difference in CE is not immediately apparent nor expected. We attribute the high CE to be linked intricately to the enhanced stability that accompanied the optimized structures. The CV plots of the 80 nm overlayer thickness sample are shown in Fig. 6b. The initial increase in current density can be attributed to an activation process that is typical for the NiO. We show reasonably stable CV peaks up to the 160th cycle, where other similar growth methods have noticeable degradation beyond the 40th cycle^26^. The degradation process for nickel oxide/hydroxide can be caused by the trapping of intercalated water when there is an imbalance in the inward and outward cycling of hydroxyl or OH^−^ ions^60^. For our optimized structures with the highly populated cracks, the increase in the material/electrolyte interfaces and better electrolyte flow can aid the out-diffusion of the OH^−^ ions that slows down the degradation process. The trapped water molecules are detrimental to the CE as they are a source for unwanted reactions that do not contribute to the optical density. Therefore, when the amount of intercalated water is reduced, CE can be increased owing to a reduction in the unwanted reaction current.

## Discussion

In summary, we have demonstrated a facile and unique method to fabricate structured NiO/Ni(OH)_2_ through template assisted fragmentation at the submicron and nanoscale. The proposed method of structuring offers a new dimension in the design and synthesis of structured metal oxides. We made use of the film overgrowth on regular pillar arrays to create mechanically weak spots that allowed for early and controlled fragmentation. Different structures of characteristic sizes, that are otherwise not achievable naturally, were obtained.

The formed structures shown can have many practical uses. Despite some attempts to use cracks in structure formation, they have previously been limited to only micrometre sizes and are far less scalable^13^,^17^,^20^,^36^. Since we have demonstrated in this work that the control of such fragmentation at the submicron and nanometre scale is possible, the low-dimensionally structured films can be an excellent base template in forming hybrid materials. The introduced approach can possibly control the extent of interaction in such hybrid materials so that their associated properties can be carefully studied. The host–guest matrix type of hybrid can be explored for materials including organics and nanoparticles, whereby they are relevant in applications such as mechanosensors^61^, catalysis^62^ and energy storage or conversion^6^. As structuring is a natural approach towards sensitivity and performance enhancement, we have demonstrated the enhanced electrochromic properties of the structured NiO in this work. The resultant structures yielded excellent electrochromic performance with high-coloration efficiency and stable cycling stability. We believe the proposed fabrication method can be extended to other electroplatable materials, other template shapes or sizes, for a variety of applications.

Our study of the crack formation at the submicron and nanoscale in the modified thin films also contributes to general studies of fragmentation. The control of fragmentation offers a method to isolate the fragmentation to certain regions of the film that can be valuable in studying the basic mechanism in fragmentation, especially for low dimensional materials. Our study shows that the early fragmentation process in this work can be attributed to a decrease in fracture toughness due to a lack of adhesion for the suspended film. The statistics of the fragmentation process also agree with current understanding on fragment size variation with thicknesses. The ability to arrest crack propagation through such modified thin films may also be an interesting concept to explore in the area of material failure analysis.

## Methods

### Patterning of substrates

ITO/glass substrates (2.5 × 2.5 cm^2^) with resistivity of 8–12 Ω per square are used as substrates. The substrates are ultrasonically cleaned in acetone and isopropyl alcohol, each for 10 min before the N1407 photoresist is spin-coated on the ITO/glass substrate. The substrate is then baked at 95 °C for 1 min before undergoing patterning via photo-exposure. In this work, LIL is chosen due to its versatility in varying pattern sizes and shapes that is necessary to examine the mechanism as intended. The samples are therefore subjected to a He–Cd laser exposure at a wavelength of 325 nm. LIL creates the regular arrays by two orthogonal exposures, through a 90° rotation of the sample for the second exposure process. Isotropic round hole (circular) patterns are achieved by keeping the exposure durations identical. Anisotropic oval hole patterns are achieved by having a 10 s difference between the two exposures. After development of the photoresist, a reactive ion etching procedure in oxygen is performed to remove any residual photoresist.

### Film growth by electroplating

Ni(OH)_2_ is cathodically electrodeposited in a solution (overall pH value of 3.5) consisting of 1.8 M Ni(NO_3_)_2_ and 0.075 M NaNO_3_ in a solvent of 50% (volume per cent) ethanol and water. The electrodeposition is accomplished using a constant voltage of −1.5 V at room temperature. After the deposition, the photoresist is removed by immersing the sample in acetone for 12 h at room temperature. The samples are subsequently dried in ambient for at least an hour (air-drying) before they are further dehydrated using a standard horizontal three-zone furnace at 300 °C for 90 min in air ambient. The conversion of Ni(OH)_2_ to NiO causes a reduction of ∼50% in the volume of the material. Since strain relief is easier in the out-of-plane direction, the change in dimension should be smaller in the in-plane direction. If we take this reduction of length to be in a 1:2 ratio, this gives an in-plane compressive strain (*ɛ*) value of ∼0.152 and represents the source of the fragmentation process in this work.

### Scanning electron microscopy

The morphology and reported thicknesses of the films are determined by SEM with a Nova NanoSEM230 at a typical acceleration voltage of 10 keV. The resolution of the SEM is ∼1.5–2.0 nm. However, enhanced emission/detection of secondary electrons at the edge of the sample can increase the inaccuracy of the cross-sectional thickness measurements to ∼5–10 nm. Practically, the dominating error in the thickness measurements can be attributed to variations across the sample area. From measurements of all samples for each growth duration, we estimate a combined deviation of <±20%. The individual deviations are highlighted both in the text and figures when necessary.

### Electrochromic performance measurements

CV measurement of the annealed Ni(OH)_2_ are carried out using a Metrohm Autolab potentiostat with a Ag/AgCl (3 M KCl) reference electrode and a platinum foil counter electrode in a 0.1 M KOH electrolyte solution. An *in-situ* monitoring of the transmittance (wavelength at 635 nm) during the CV cycling is performed. The transmittance monitoring setup consists of a Thorlab laser and silicon photodiode.

### Data availability

All the relevant data that supports the findings and conclusion of this work are available from the corresponding authors on request.

## Additional information

**How to cite this article:** Guo, L. *et al*. Ordered fragmentation of oxide thin films at submicron scale. *Nat. Commun.* **7,** 13148 doi: 10.1038/ncomms13148 (2016).

## Supplementary Material

Supplementary Information Supplementary Figures 1 - 6, Supplementary Table 1, Supplementary Notes 1 - 6 and Supplementary References

## Figures and Tables

**Table 1:** Comparison of reported electrochromic performance.

**λ (nm)**	**Material structure**	**|ΔOD|**	**|CE|(cm**^**2**^**C**^**−1**^**)**	**References**
630	Nanostructure	2.1	40–47	Scherer *et al*.^8^
630	Nanostructure	1.06	43.3	Patil *et al*.^58^
635	Nanostructure	0.62	50.5	Guo *et al*.^26^
635	Nanostructure	0.99	72.2	This study
635	Thin film	0.23	34.9	This study

Comparison of the optical density modulation (Δ*OD*) and coloration efficiency (CE) of NiO/Ni(OH)_2_ based structures with other reports^8^,^26^,^58^.

## References

[b1] Liu C. . An all-in-one nanopore battery array. Nat. Nanotechnol. 9, 1031–1039 (2014). 25383515 10.1038/nnano.2014.247

[b2] Maier J. Nanoionics: ion transport and electrochemical storage in confined systems. Nat. Mater. 4, 805–815 (2005). 16379070 10.1038/nmat1513

[b3] Honciuc A. . Controlling the adsorption kinetics via nanostructuring: Pd nanoparticles on TiO_2_ nanotubes. Langmuir 26, 14014–14023 (2010). 20698520 10.1021/la102163a

[b4] Taberba P. L., Mitra S., Poizot P., Simon P. & Tarascon J. M. High rate capabilities Fe_3_O_4_-based Cu nano-architectured electrodes for lithium-ion battery applications. Nat. Mater. 5, 567–573 (2006). 16783360 10.1038/nmat1672

[b5] Shi Y. . Ordered mesoporous metallic MoO_2_ materials with highly reversible lithium storage capacity. Nano Lett. 9, 4215–4220 (2009). 19775084 10.1021/nl902423a

[b6] Wang H. . Hybrid device employing three-dimensional arrays of MnO in carbon nanosheets bridges battery-supercapacitor divide. Nano Lett. 14, 1987–1994 (2014). 24617337 10.1021/nl500011d

[b7] Wei D. . A nanostructured electrochromic supercapacitor. Nano Lett. 12, 1857–1862 (2012). 22390702 10.1021/nl2042112

[b8] Scherer M. R. J. & Steiner U. Efficient electrochromic devices made from 3D nanotubular gyroid network. Nano Lett. 13, 3005–3010 (2013). 23205700 10.1021/nl303833h

[b9] Birnkrant M. J. . Layer-in-layer hierarchical nanostructures fabricated by combining holographic polymerization and block copolymer self-assembly. Nano Lett. 7, 3128–3133 (2007). 17854228 10.1021/nl071673j

[b10] Xia Y., Yin Y., Lu Y. & McLellan J. Template-assisted self-assembly of spherical colloids into complex and controllable structures. Adv. Funct. Mater. 13, 907–918 (2003).

[b11] Cheng J. Y., Ross C. A., Smith H. I. & Thomas E. L. Templated self-assembly of block copolymers top-down helps bottom-up. Adv. Mater. 18, 2505–2521 (2006).

[b12] Naureen S., Sanatinia R., Shahid N. & Anand S. High optical quality InP-based nanopillars fabricated by a top-down approach. Nano Lett. 11, 4805–4811 (2011). 21942530 10.1021/nl202628m

[b13] Han W. & Lin Z. Drying-mediated assembly of colloidal nanoparticles into large-scale microchannels. ACS Nano 7, 6079–6085 (2013). 23730974 10.1021/nn401885f

[b14] Kerr R. & Thomas I. Song Dynasty Ceramics V&A Publications (2004).

[b15] Lahlil S., Xu J. & Li W. Influence of manufacturing parameters on the crackling process of ancient Chinese glazed ceramics. J. Cult. Herit. 16, 401–412 (2015).

[b16] Zhou J. . Study on the formation mechanism and preparation of ice crackle celadon. J. Synth. Cryst. 40, 1076–1082 (2011).

[b17] Pauchard L., Elias F., Boltenhagen P., Cebers A. & Bacri J. C. When a crack is oriented by a magnetic field. Phys. Rev. E 77, 021402 (2008). 10.1103/PhysRevE.77.021402 18352026

[b18] Khatun T., Dutta T. & Tarafdar S. Crack formation under an electric field in droplets of laponite gel memory effect and scaling relations. Langmuir 29, 15535–15542 (2013). 24308830 10.1021/la404297k

[b19] Graham M. & Morris S. Development and geometry of isotropic and directional shrinkage-crack patterns. Phys. Rev. E 61, 6950–6957 (2000). 10.1103/physreve.61.6950 11088387

[b20] Nam K. H., Park H. II & Ko S. H. Patterning by controlled cracking. Nature 485, 221–224 (2012). 22575963 10.1038/nature11002

[b21] Kim B. C. . Guided Fracture of films on soft substrates to create micro/nano-feature arrays with controlled periodicity. Sci. Rep. 3, 3027 (2013). 24149668 10.1038/srep03027 PMC3805969

[b22] Kim M., Ha D. & Kim T. Cracking-assisted photolithography for mixed-scale patterning and nanofluidic applications. Nat. Commun. 6, 6247 (2015). 25692794 10.1038/ncomms7247

[b23] Li H. . Amorphous nickel hydroxide nanospheres with ultrahigh capacitance and energy density as electrochemical pseudocapacitor materials. Nat. Commun. 4, 1894 (2013). 23695688 10.1038/ncomms2932 PMC3674274

[b24] Yang Y. . Hydrothermally formed three-dimensional nanoporous Ni(OH)_2_ thin-film supercapacitors. ACS Nano 8, 9622–9628 (2014). 25198148 10.1021/nn5040197

[b25] Ji J. . Nanoporous Ni(OH)_2_ thin film on 3D ultrathin graphite foam for asymmetric supercapacitor. ACS Nano 7, 6237–6243 (2013). 23758135 10.1021/nn4021955

[b26] Guo L., Ren Y., Liu J., Chiam S. Y. & Chim W. K. Nanostructuring of nickel hydroxide via a template solution approach for efficient electrochemical devices. Small 10, 2611–2617 (2014). 24634166 10.1002/smll.201303889

[b27] Pang H., Lu Q., Li Y. & Gao F. Facile synthesis of nickel oxide nanotubes and their antibacterial, electrochemical and magnetic properties. Chem. Commun. 48, 7542–7544 (2009). 10.1039/b914898a 20024273

[b28] Xia X. . High-quality metal oxide core/shell nanowire arrays on conductive substrates for electrochemical energy storage. ACS Nano 6, 5531–5538 (2012). 22545560 10.1021/nn301454q

[b29] Domokos G., Kun F., Sipos A. A. & Szabo T. Universality of fragment shapes. Sci. Rep. 5, 9147 (2015). 25772300 10.1038/srep09147 PMC4360630

[b30] Li L., Zhang Z., Zhang P., Wang Z. & Zhang Z. Controllable fatigue cracking mechanisms of copper bicrystals with a coherent twin boundary. Nat. Commun. 5, 3536 (2014). 24667520 10.1038/ncomms4536

[b31] Qin Z., Pugno N. M. & Buehler M. J. Mechanics of fragmentation of crocodile skin and other thin films. Sci. Rep. 4, 4966 (2014). 24862190 10.1038/srep04966 PMC4034009

[b32] Meakin P. A simple model for elastic fracture in thin films. Thin Solid Films 151, 165–190 (1987).

[b33] Sen D. & Buehler M. J. Structural hierarchies define toughness and defect-tolerance despite simple and mechanically inferior brittle building blocks. Sci. Rep. 1, 35 (2011). 22355554 10.1038/srep00035 PMC3216522

[b34] Yao H., Xie Z., He C. & Dao M. Fracture mode control: a bio-inspired strategy to combat catastrophic damage. Sci. Rep. 5, 8011 (2011). 10.1038/srep08011 PMC4306140 25619564

[b35] Ritchie R. O. The conflicts between strength and toughness. Nat. Mater. 10, 817–822 (2011). 22020005 10.1038/nmat3115

[b36] Green D. J., Tandon R. & Sglavom V. M. Crack arrest and multiple cracking in glass through the use of designed residual stress profiles. Science 283, 1295–1297 (1999). 10037593 10.1126/science.283.5406.1295

[b37] Lange F. F. Chemical solution routes to single-crystal thin films. Science 273, 903–909 (1996). 8688066 10.1126/science.273.5277.903

[b38] Roeder R. K. & Slamovich E. B. Assessment of the critical thickness and fracture toughness of thin metal-organic precursor films. Ceram. Trans. 83, 375–382 (1998).

[b39] Kitsunezaki S. Crack growth in drying paste. Adv. Powder Technol. 22, 311–318 (2011).

[b40] Groisman A. & Kaplan E. An experimental study of cracking induced by desiccation. Europhys. Lett. 26, 415–420 (1994).

[b41] Griffith A. A. The phenomena of rupture and flow in solids. Philos. Trans. R. Soc. Lond. A 221, 163–198 (1921).

[b42] Han S. Y. . The growth mechanism of nickel oxide thin films by room-temperature chemical bath deposition. J. Electrochem. Soc. 153, C382–C386 (2006).

[b43] Xia X. . Electrochromic properties of porous NiO thin films prepared by CBD. Sol. Energy Mater. Sol. Cells 92, 628–633 (2008).

[b44] Lee W. P. & Routh A. F. Why do drying films crack? Langmuir 20, 9885–9888 (2004). 15518466 10.1021/la049020v

[b45] Ngo A. T., Richardi J. & Pileni M. P. Cracks in magnetic nanocrystal films: do directional and isotropic crack patterns follow the same scaling law? Nano Lett. 8, 2485–2489 (2008). 18630886 10.1021/nl801501y

[b46] Leung K. & Neda Z. Pattern formation and selection in quasistatic fracture. Phys. Rev. Lett. 85, 662–665 (2000). 10991365 10.1103/PhysRevLett.85.662

[b47] Smith M. I. & Sharp J. S. Effects of substrate constraint on crack pattern formation in thin films of colloidal polystyrene particles. Langmuir 27, 8009–8017 (2011). 21650173 10.1021/la2000624

[b48] Chen D. Z. . Nanometallic glasses: size reduction brings ductility, surface state drives its extent. Nano Lett. 13, 4462–4468 (2013). 23978318 10.1021/nl402384r

[b49] Krzyzanowski M., Beynon J. H. & Farrugia C. J. Oxide Scale Behavior in High Temperature Metal Processing Wiley-VCH (2010).

[b50] Street R. & Lewis B. Anomalous variation of Young's Modulus of antiferromagnetics at the Neel point. Nature 168, 1036–1037 (1951).

[b51] Fasaki I., Koutoulaki A., Kompitsas M. & Charitidis C. Structural, electrical and mechanical properties of NiO thin films grown by pulsed laser deposition. Appl. Surf. Sci. 257, 429–433 (2010).

[b52] Gogotsi G. A. Fracture toughness of ceramics and ceramic composites. Ceram. Int. 29, 777–784 (2003).

[b53] Tromans D. & Meech J. A. Fracture toughness and surface energies of minerals: theoretical estimates for oxides sulphides silicates and halides. Miner. Eng. 15, 1027–1041 (2002).

[b54] Bradt R. C., Munz D., Sakai M., Shevchenko V. Y. & White K. W. Crack-Mircostructure Interaction, R-curve Behavior, Environmental Effects in Fracture and Standardization Vol. 13, Springer Series (2002).

[b55] Li J., Forberg S. & Hermansson L. Evaluation of the mechanical properties of hot isostatically pressed titania and titania-calcium phosphate composites. Biomaterials 12, 438–440 (1991). 1888813 10.1016/0142-9612(91)90015-3

[b56] Schneller T., Waser R., Kosec M. & Payne D. Chemical Solution Deposition of Functional Oxide Thin Films Springer (2009).

[b57] Monk P., Mortimer R. & Rosseinsky D. Electrochromism and Electrochromic Devices Cambridge University Press (2007).

[b58] Patil R. A. . Efficient electrochromic properties of high-density and large-area arrays of one-dimensional NiO nanorods. Sol. Energy Mater. Sol. Cells 112, 91–96 (2013).

[b59] Subrahmanyam A. & Karuppasamy A. Optical and electrochromic properties of oxygen sputtered tungsten oxide (WO_3_) thin films. Sol. Energy Mater. Sol. Cells 91, 266–274 (2007).

[b60] Ren Y. . The coloration and degradation mechanism of electrochromic nickel oxide. Sol. Energy Mater. Sol. Cells 116, 83–88 (2013).

[b61] Kang D. . Ultrasensitive mechanical crack-based sensor inspired by the spider sensory system. Nature 516, 222–226 (2014). 25503234 10.1038/nature14002

[b62] Zhang P., Qiao Z., Jiang X., Veith G. A. & Dai S. Nanoporous ionic organic networks stabilizing and supporting gold nanoparticles for catalysis. Nano Lett. 15, 823–828 (2015). 25625306 10.1021/nl504780j

