# Supplementary material for: Ordered fragmentation of oxide thin films at submicron scale
Source: Nat Commun. 2016 Oct 17;7:13148. doi: 10.1038/ncomms13148 (PMC5071645; doi:10.1038/ncomms13148)
Supplement: Supplementary Information — Supplementary Figures 1 - 6, Supplementary Table 1, Supplementary Notes 1 - 6 and Supplementary References [file ncomms13148-s1.pdf]

Supplementary Figures

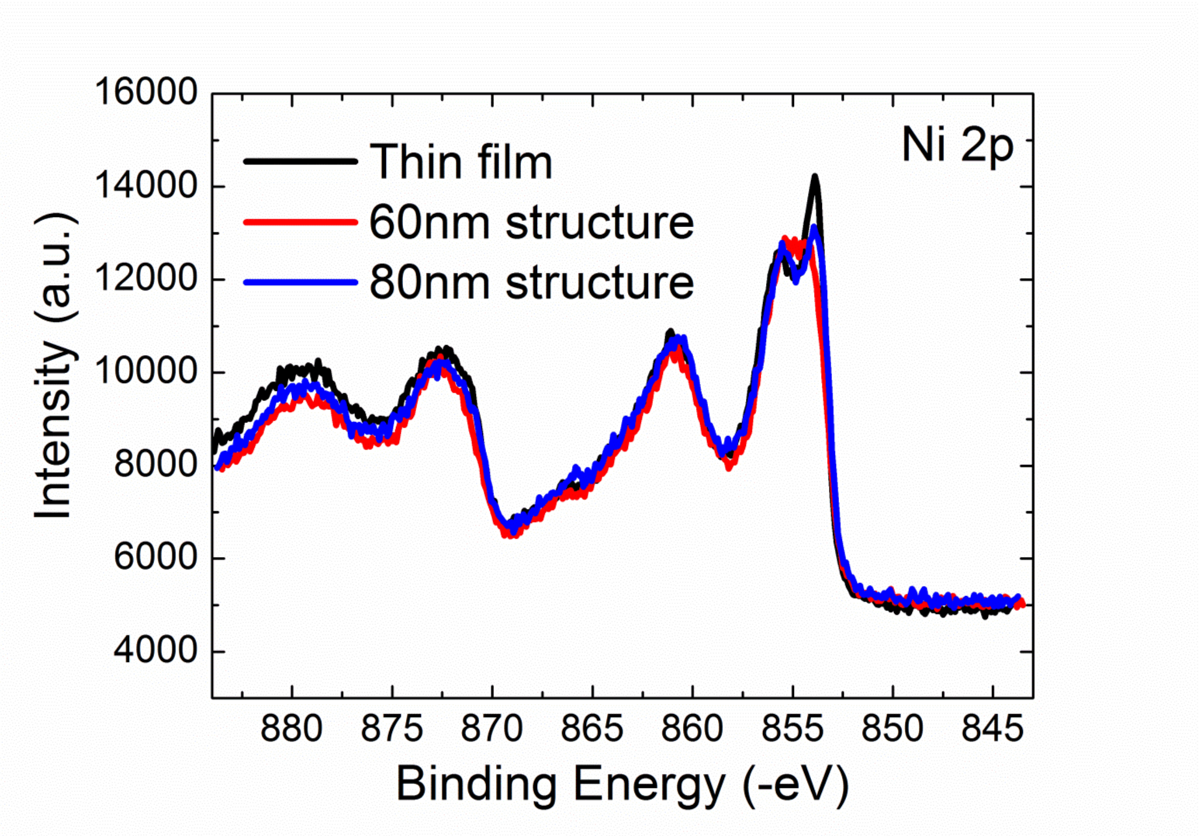

Supplementary Figure 1. X-ray photoelectron spectroscopy Ni 2p spectra for thin film and as plated structures with indicated overlayer thicknesses, after thermal annealing.

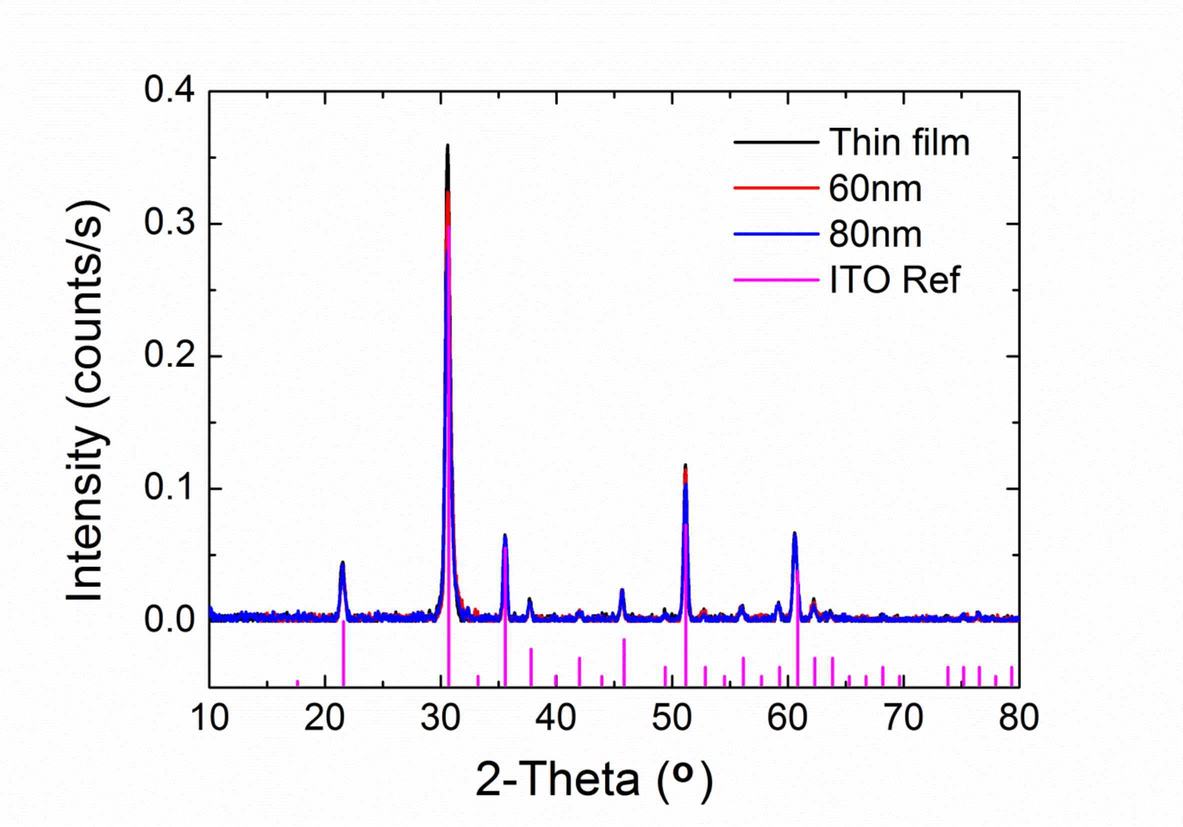

Supplementary Figure 2. X-ray diffraction (XRD) spectra of thin and structured films with the indicated thicknesses, after thermal treatment at 300ºC. The reference for ITO coating is also shown.

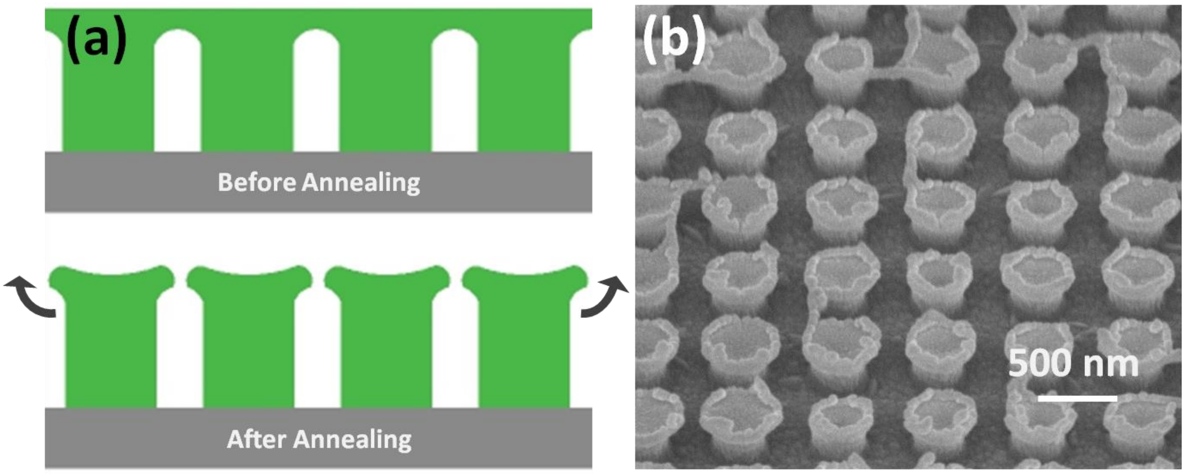

Supplementary Figure 3. (a) Schematic showing the crinkle effect of a 80nm film before and after annealing. (b) Actual SEM image of pillars with crinkle effects after annealing.

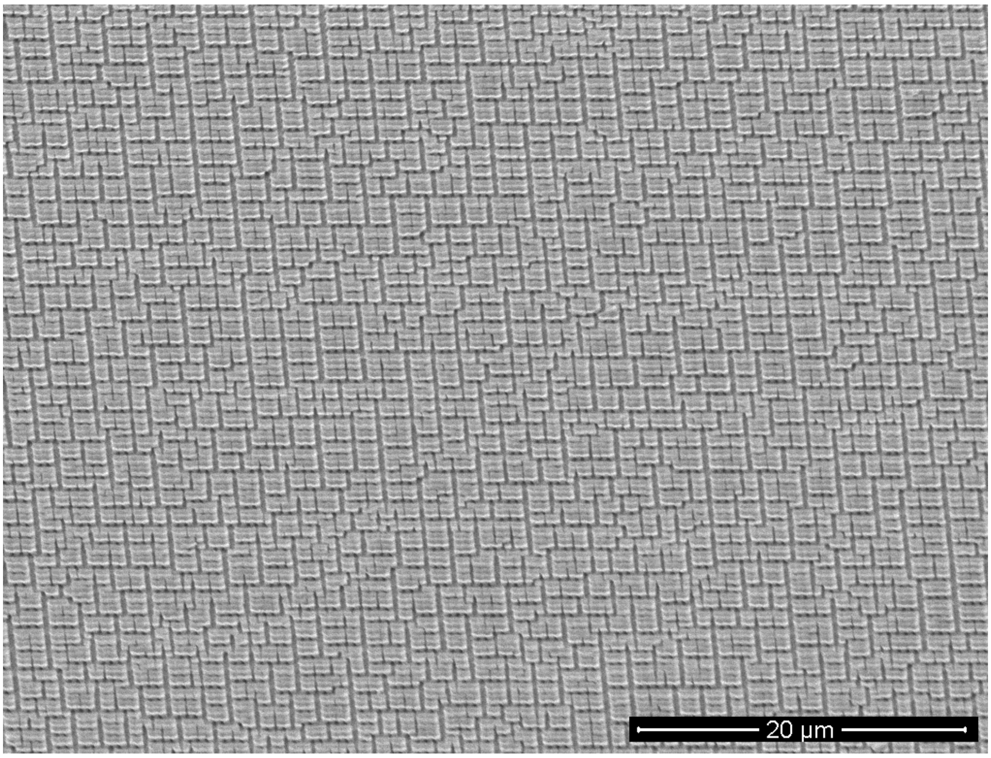

Supplementary Figure 4. Low magnification (4000×) SEM image of formed patterns after the drying/fragmentation process for a 120nm film plated through round holes.

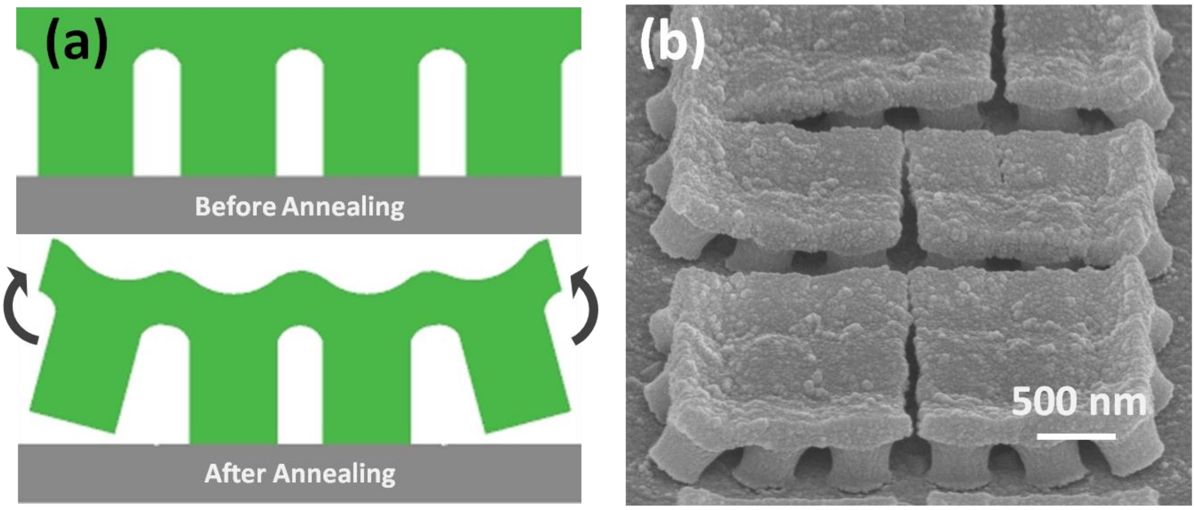

Supplementary Figure 5. (a) Schematic showing the crinkle effect of a 140nm film before and after annealing. (b) Actual SEM image of pillars fragments with crinkle effects after annealing.

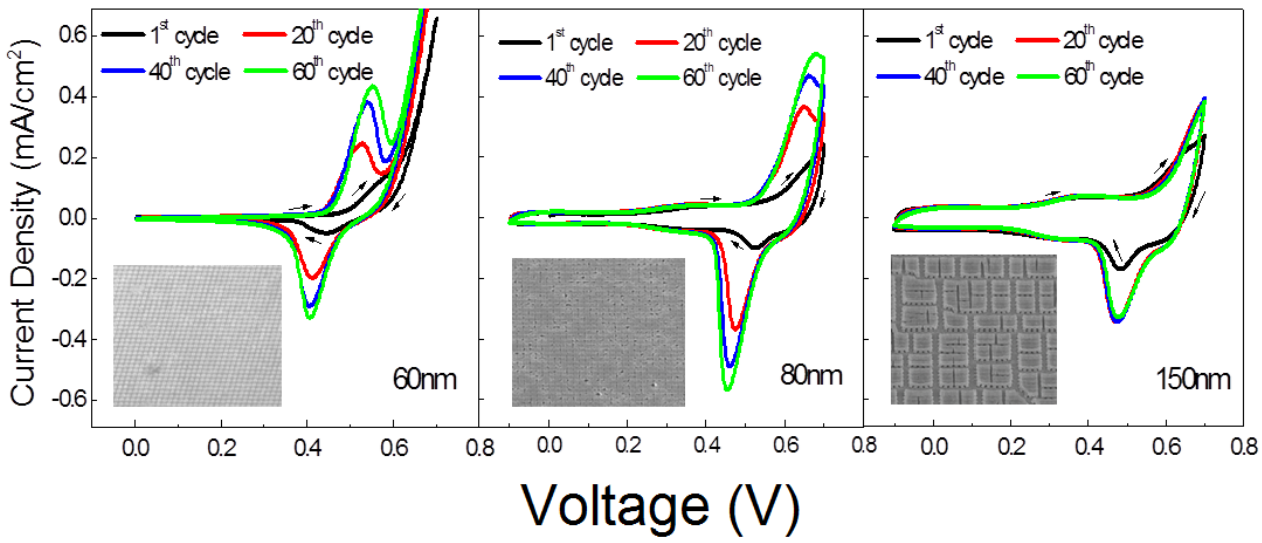

Supplementary Figure 6. Cyclic-voltammetry (CV) of structured films using circular patterns for indicated overlayer thickness of 60nm, 80nm and 150nm. The inset shows SEM images of the cycled samples.

Supplementary Table 1. Quantification of Ni/O ratio using fitted Ni 2p and O 1s fitted peaks. The binding energies shown are for the main line. The peaks were fitted after a Shirley background subtraction, using a Voigt profile.

Sample	Ni 2p (%)	Ni $2p_{3/2}$ (eV)	O 1s(%)	O 1s (eV)	Ratio Ni/O
Thin film	34.59	853.83	65.41	529.32	0.53
60nm structure	33.66	853.78	66.33	529.31	0.51
80nm structure	34.35	853.88	65.65	529.39	0.52

Supplementary Note 1

In Supplementary Figure 1, X-ray photoelectron spectroscopy (XPS) was performed using the ThermoScientific Theta Probe XPS system equipped with a concentric hemispherical energy analyser. The monochromatic Al *Kα* is used as the radiation source. Quantitative analysis was performed using a Shirley background subtraction before a least-square-error fit was performed with a mixture of Gaussian-Lorentzian lineshapes. The overlay of the spectra for Ni 2p, for the as-plated thin film and the as-plated structures after annealing, are shown in Supplementary Figure 1. The split observed around 1.5 eV from the main line (~853.8 eV) is a final-state effect due to non-local screening [1]. It is therefore less strongly observed for structured films with reduced number of nearest neighbours [2]. Despite this difference, the chemical composition of the film remains similar, as shown in the fitted summary in Supplementary Table 1. We note that for the structured sample, signals from the indium tin oxide (ITO) substrate can be detected from the cracks and fragmentation. Therefore, the presented O 1s data are after subtraction of the oxygen contribution from the ITO substrate. The Ni/O ratios obtained are reasonably similar with a range from 0.51-0.53. The annealed film has a stoichiometry closer to NiO_2.

Supplementary Note 2

The crystallinity of the annealed thin films and structured samples shown in Supplementary Figure 2 were examined using X-ray diffraction (XRD) 2-theta scans using a GADDS XRD system equipped with Cu Kα radiation. The resulting spectra are shown in Supplementary Figure 2, together with the reference data of In_2O_3 representing the ITO coating (JCPDS-ICDD 06-416). It can be seen that aside from the peaks from the indium oxide, no diffraction peaks from the thin film or structured film can be observed. This shows that the film is amorphous in nature even after the thermal treatment at 300ºC.

Supplementary Note 3

The schematic in Supplementary Figure 3(a) shows the suspended portion of the film that remains after breakage during annealing. Upward warping is seen after dehydration as shown in Supplementary Figure 3(b). This phenomenon can be understood from preferential strain relief. This is because further strain relief can be achieved by direct volume reduction without the need for formation of more cracks. The faster loss of water from the surface can result in rapid shrinkage of exposed surfaces that causes the eventual upward warping of the suspended portion. This upward crinkling effect can also be aided by the anchoring pillar in providing some physical inhibition for a downward movement.

Supplementary Note 4

The low magnification SEM image in Supplementary Figure 4 shows the formation of fragments over a large area after the drying process for a 120nm film plated through round holes. The distinct crack formation yielded interesting mosaic-like patterns across the surface. The image shows the presence of some randomness in the fragment creation while maintaining characteristic sizes across the sample surface.

Supplementary Note 5

For the larger fragments, a similar warping mechanism means the detachment of the pillars at the edges after annealing, as shown in the schematic in Supplementary Figure 5(a) and the SEM image in Supplementary Figure 5(b). There are competing forces for crack creation and warping of the whole structure in this case. The resultant SEM shows a structure whereby the concurrent warping and crack formation for the large structure possibly occurred. This yielded warping only on the sides of the structure with crack formation in the middle of the structure.

Supplementary Note 6

The cyclic-voltammetry (CV) of structured film with the respective indicated thickness is shown in Supplementary Figure 6 together with their SEM images in the inset as a representation of the structures. The CV is shown for the first 60 cycles. The anodic and cathodic potential are similarly shaped although it can be observed that the current density differs with the highest current density obtained for the 80nm overlayer thickness. As the thicknesses increases, there is a slight increase in the series resistance and hence we see a shift of the CV curves towards a higher potential. This increase is ~0.07V across the three samples.

Supplementary References

[1] Veenendaal, M. A. van & Sawatzky, G. A. Nonlocal screening effects in 2*p* X-ray photoemission spectroscopy core-level line shapes of transition metal compounds. *Phys. Rev. Lett.* **70**, 2459-2462 (1993).

[2] Alders, D. , Voogt, F. C. , Hibma, T. & Sawatzky G. A. Nonlocal screening effects in 2*p* X-ray photoemission spectroscopy of NiO(100). *Phy. Rev. B.* **54**, 7716-7719 (1996).
